# The trophic niche of subterranean populations of *Speleomantes italicus*

**DOI:** 10.1038/s41598-022-21819-8

**Published:** 2022-10-29

**Authors:** Enrico Lunghi, Fabio Cianferoni, Claudia Corti, Yahui Zhao, Raoul Manenti, Gentile Francesco Ficetola, Giorgio Mancinelli

**Affiliations:** 1 Key Laboratory of the Zoological Systematics and Evolution, Institute of Zoology, Chinese Academy of Sciences, Beijing, China; 2 Dipartimento di Medicina clinica, sanità pubblica, scienze della vita e dell’ambiente (MESVA), University of L’Aquila, Coppito, L’Aquila Italy; 3 Zoologia, “La Specola”, Museo di Storia Naurale, Università degli Studi di Firenze, Florence, Italy; 4 Natural Oasis, Prato, Italy; 5 Unione Speleologica Calenzano, Calenzano, Florence, Italy; 6 Istituto di Ricerca Sugli Ecosistemi Terrestri (IRET), Consiglio Nazionale delle Ricerche (CNR), Sesto Fiorentino, Florence Italy; 7 Dipartimento di Scienze e Politiche Ambientali, Università degli Studi di Milano, Milano, Italy; 8 Laboratorio di Biologia Sotterranea “Enrico Pezzoli”, Parco Regionale del Monte Barro, Galbiate, Italy; 9 Laboratoire d’Écologie Alpine (LECA), Université Grenoble Alpes, CNRS, Grenoble, France; 10 Dipartimento di Scienze e Tecnologie Biologiche ed Ambientali (DiSTeBA), Università del Salento, Lecce, Italy; 11 Istituto per le Risorse Biologiche e le Biotecnologie Marine (IRBIM), Consiglio Nazionale delle Ricerche (CNR), Lesina, Foggia Italy; 12 CoNISMa, Consorzio Nazionale Interuniversitario per le Scienze del Mare, Roma, Italy

**Keywords:** Ecology, Behavioural ecology

## Abstract

The determination of a species trophic niche can clarify its functional role within a food web and how prey resources are used in relation with the spatial and temporal variability of environmental conditions. This information may result particularly useful for the implementation of conservation plans of endangered species having a cryptic behaviour or living in places difficult to be surveyed. Here we present the first long-term study on the trophic niche of the Italian cave salamander *Speleomantes italicus*, a strictly protected facultative cave species that seasonally exploits surface environments (e.g., forested areas) as well as both natural and artificial subterranean environments. We analysed the diet variation of six populations of *S. italicus* inhabiting natural caves, surveyed 24 times in a full year. During the surveys, all sampled individuals were subjected to stomach flushing and the ingested prey were identified and enumerated; furthermore, salamanders’ body condition was also evaluated. The results of the analyses provided the first comprehensive, year-round assessment of the diet for a *Speleomantes* species. Remarkable divergences in terms of trophic niche and body condition were observed between the studied populations. We found a discrepancy in the foraging activity of the populations located in different areas; specifically, the individuals that experienced sub-optimal microclimatic conditions poorly performed in foraging. Furthermore, we found temporal and spatial variability in the body condition of individuals. Our study highlighted a remarkably high spatial and temporal divergence in the trophic habits of conspecific populations, a feature that may represent one of the major factors promoting the variability of multiple population traits.

## Introduction

The trophic niche of a species can be defined as the n-dimensional hyper-volume representing the role in a food web of a particular species, and represents a key component of its ecological niche^1^. The study of the diet of a species, its trophic position in the ecosystem, and the ecology of its food resources are of pivotal importance to correctly understand species’ ecology and to plan conservation strategies for those on the brink of extinction^2–6^. However, studying a species’ diet may be challenging. Species may have complex foraging activities (e.g., using particular techniques or systematically switching to different foraging areas^7,8^) or be characterized by a high temporal variability in their diet composition^9,10^; therefore dietary data with a low spatial and temporal resolution may not be sufficient to obtain reliable information^11,12^. Indeed, species diet is often studied only over short periods^13–15^, seldom including observations replicated over different seasons^16–18^. Only rarely surveys involve a systematic and regular collection data over long periods (e.g.,^19–22^). If the trophic niche of species changes through time, for instance because of differences in requirements or in food availability, sporadic observations would hamper a correct assessment of the trophic role of a species. In some circumstances the cryptic behaviour of the species^23,24^ or the difficulties in accessing the environment in which the species live^25,26^ may represent further limitations. In these cases, well-designed non-lethal methods can help filling these gaps without compromising the survival of the examined individuals^27,28^.

Here we focused on the diet of salamanders belonging to the genus *Speleomantes* (also referred as *Hydromantes*; see for discussion^29^). Commonly known as European cave salamanders, they are the only plethodontids occurring in Europe^30^; all the species are strictly protected by national and international laws^31,32^. Five of the eight species of *Speleomantes* (*S. flavus*, *S. supramontis*, *S. imperialis*, *S. sarrabusensis*, *S. genei*) are endemic to Sardinia (Italy), while the other three (*S. strinatii*, *S. ambrosii*, *S. italicus*) are distributed along the Apennines and Maritime Alps in continental Italy; one (*S. strinatii*) extends from north-western Italy over a small area of French Provence^30^. These salamanders are facultative cave species, as they choose to live in specific subterranean environments that represent a safe refuge both from unsuitable climatic conditions and from potential predators^33,34^, but feed inside as well as outside of the caves^35,36^. Cave salamanders usually exploit small cracks to maintain homeostasis and avoid potential disturbances^37–39^, but these environments often have difficult accessibility, and this makes the assessment of their biological and ecological characteristics challenging^27,40^. *Speleomantes* are generalist predators that prey on a large number of subterranean and epigean species^41,42^. The direct observation of their predation activity is arduous as salamanders mostly feed in the dark, hidden under logs or stones, and because of the very high speed with which they capture prey with their tongue^43,44^.

**Figure 1:**
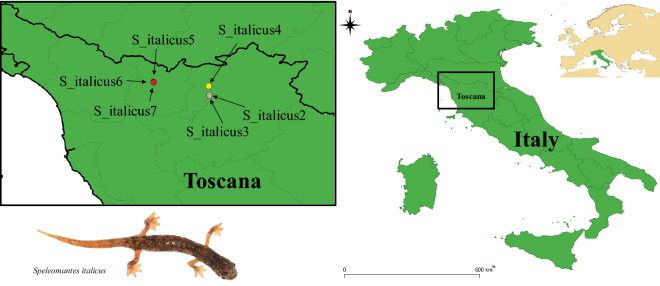
The map of the study area indicating the location of the studied caves. Three caves were located in the municipality of Pistoia (red circles; S_italicus5, S_italicus6, S_italicus7), 1 in the municipality of Vaiano (yellow circle; S_italicus4) and 2 in the municipality of Prato (grey circles; S_italicus2, S_italicus3). No detailed information on sampled locations are provided to ensure species protection^84^. In the bottom-left corner an individual of *Speleomantes italicus* (the image is taken from the dataset published by Lunghi et al.^83^, as well as the populations code). Map was built using the free software QGis v. 3.8.3 (https://qgis.org/en/site/).

Long-term studies on species trophic niche are very scarce across animal species, but still very important to properly understand species requirements. The trophic niche of *Speleomantes* has been studied only through single surveys carried out in spring and/or autumn, periods characterized by particularly suitable climatic conditions allowing them to forage outside subterranean environments^35,36,45,46^. Despite *Speleomantes* show strong seasonal variation in habitat use^47^, no studies on the *Speleomantes*’ diet have covered the variation that could occur over a full year, leaving many aspects of their trophic habits unknown. Using as a model six subterranean populations of *S. italicus* (Table 1, Fig. 1), we aimed to test the following hypotheses: (*i*) do *Speleomantes* forage throughout the year or only when climatic conditions allow to exploit epigeous environments? It is not known if *Speleomantes* remain completely inactive when environmental conditions exceed their tolerance limits (e.g., in summer and winter)^37,48^ or if they can forage all year round thanks to the stable microclimate found in subterranean environments^47^. These salamanders exploit subterranean environments mainly to avoid unsuitable climatic conditions^49^, and during the harsh seasons (summer and winter) they move to deeper cave areas where the microclimate remains suitable, but the availability of prey is scarce^36,47,50^. (*ii*) If *Speleomantes* forage through the whole year, does their diet change accordingly? A recent study suggested that *Speleomantes*’ diet may change between spring and summer, mainly because of the seasonal variation of ecological opportunities^16,51^; however, without information on their yearly trophic activity is it not possible to distinguish between actual changes of the diet, and potential effect of stochastic factors. (*iii*) Does body condition change across seasons? Considering the potential variation in the abundance and diversity of prey consumed during the different seasons^16^, one can expect a variation in the body condition of salamanders throughout the year related to the intensity of the foraging activity of the salamanders which, in turn, depends on climatic conditions^36^. Furthermore, strong inter-individual competition for access to the most profitable resources can occur in high-density populations^52,53^ and this can negatively affect the overall body condition of the population^36,54^.

**Table 1:** Summary of the data related to the studied cave populations (retrieved from^74^).

Population	Latitude	Longitude	Elevation	Municipality	Total captured individuals	Max density	Total recognized prey
S_italicus2	43.92	11.16	699	Prato	115	5.29	961
S_italicus3	43.92	11.16	715	Prato	190	0.31	2157
S_italicus4	43.98	11.16	492	Vaiano	133	0.37	644
S_italicus5	44	10.82	948	Pistoia	6	0.1	73
S_italicus6	44	10.82	853	Pistoia	9	0.15	54
S_italicus7	44	10.82	850	Pistoia	42	0.41	288

For each population we show: the population code (following^83^); latitude, longitude and elevation (m a.s.l.) of the cave entrance; Municipality (where the cave is located); the total number of captured individuals (excluding recaptures); the maximum density of salamanders (individuals/m^2^; calculated considering the maximum captured individuals and the surveyed area); the total number of recognized prey through the whole study (i.e., including recaptures).

## Results

We detected salamanders in every month except January; the highest number of active individuals was observed in May, the month in which we collected the largest number of prey consumed (Table 2). The estimated maximum density widely diverged between the studied populations, being the highest in S_italicus2 and the lowest in S_italicus5 (Table 1). Although the overall diet of *S. italicus* populations included 31 different prey categories, salamanders mostly fed on flies (Diptera; 69.83%), adult beetles (Coleoptera; 14.84%, of which 2.24% Staphylinidae) and spiders (Araneae; 2.94%), with a marginal contribution (12.39%) from the other 27 groups (Table 2). Detailed monthly information on captured salamanders and prey consumed is shown in Table 2.

**Table 2:** Summary of the monthly prey consumed by individuals from the six subterranean populations of *Speleomantes italicus*.

Population		January	February	March	April	May	June	July	August	September	October	November	December
S_italicus2	N individuals	0	3	2	8	52	20	8	6	17	37	2	3
	N prey		25	5	21	395	121	23	15	67	237	31	21
	Major contributors		Diptera (96%)	Diptera (100%)	Dipera (52%)	Diptera (77%)	Diptera (97%)	Diptera (100%)	Diptera (80%)	Coleoptera (58%)	Coleoptera (65%)	Coleoptera_Staphylinidae (90%)	Diptera (95%)
					Coleoptera_larva (10%)	Coleoptera (22%)			Coleoptera (20%)	Diptera (37%)			
					Araneae (10%)								
					Isopoda (10%)								
S_italicus3	N individuals	0	6	4	11	93	57	14	2	30	45	11	
	N prey		29	11	41	872	521	62	8	175	317	112	5
	Major contributors		Polydesmida (45%)	Diptera (36%)	Diptera (46%)	Diptera (80%)	Diptera (87%)	Diptera (81%)	Diptera (100%)	Diptera (77%)	Diptera (50%)	Coleoptera_Staphylinidae (21%)	Diptera (40%)
			Diptera (28%)		Coleoptera_Staphylinidae (12%)	Coleoptera (11%)				Coleoptera (12%)	Coleoptera (22%)	Symphypleona (14%)	Symphypleona (20%)
												Polydesmida (10%)	Poduromorpha (20%)
												Entomobryomorpha (10%)	Entomobryomorpha (20%)
												Diptera (10%)	
S_italicus4	N individuals	0	0	4	7	59	35	20	12	9	9	3	1
	N prey			20	20	247	185	60	22	10	51	27	2
	Major contributors			Diptera (90%)	Diptera (50%)	Diptera (96%)	Diptera (99%)	Diptera (98%)	Diptera (95%)	Diptera (80%)	Hymenoptera_Formicidae (20%)	Diptera_larva (30%)	Araneae (50%)
				Orthoptera (10%)	Araneae (25%)					Araneae (10%)	Araneae (18%)	Symphypleona (22%)	Diptera (50%)
					Coleoptera (10%)					Isopoda (10%)	Diptera (10%)	Orthoptera (15%)	
												Diptera (11%)	
S_italicus5	N individuals	0	0	0	0	5	0	0	0	0	1	0	0
	N prey					56					17		
	Major contributors					Diptera (70%)					Hymenoptera (82%)		
											Araneae (18%)		
S_italicus6	N individuals	0	0	0	1	7	0	0	0	0	1	0	1
	N prey				8	31					8		7
	Major contributors				Diptera (38%)	Diptera (84%)					Araneae (25%)		Diptera (86%)
					Araneae (25%)						Polydesmida (25%)		Diptera_larva (14%)
					Polydesmida (13%)						Blattodea (13%)		
					Hemiptera (13%)						Hymenoptera_Formicidae (13%)		
					Trichoptera_larva (13%)						Coleoptera (13%)		
											Archaeognatha (13%)		
S_italicus7	N individuals	0	2	0	7	12	6	2	0	2	4	3	10
	N prey		14		22	80	33	8		2	13	58	58
	Major contributors		Diptera (93%)		Diptera (55%)	Diptera (73%)	Diptera (97%)	Diptera (100%)		Diptera (100%)	Diptera (46%)	Symphypleona (26%)	Diptera (88%)
					Araneae (18%)						Araneae (23%)	Araneae (17%)	
					Coleoptera (14%)							Diptera (17%)	
												Diptera_larva (12%)	

The table shows the following monthly data for each populations: number of individuals undergoing stomach flushing, total number of prey recognized from stomach contents, the Major contributors to population diet (only prey categories which contribution is ≥ 10% are shown).

**Figure 2:**
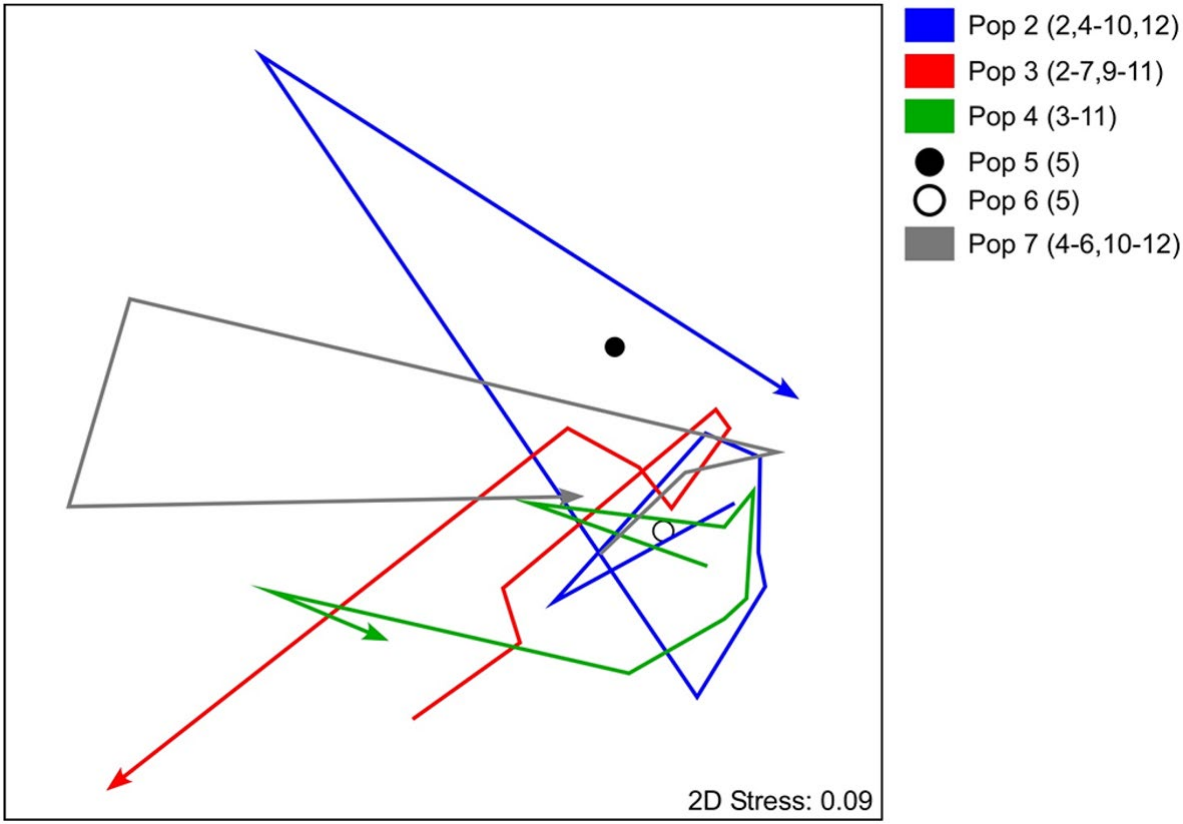
nMDS plot of temporal trajectories in dietary variations among the six populations of *Speleomantes italicus* under analysis; in the caption, the months when individuals were analysed are reported in parentheses.

The frequency of empty stomachs was significantly different between the populations (Post-hoc comparison test; *χ* = 76.31, df = 7, *P* < 0.001) and between months (*χ* = 89.75, df = 12, *P* < 0.001). Empty stomachs were less frequent in May and June.

The number of prey items consumed was significantly different between populations (*F*_*5,798.92*_ = 14.68, *P* < 0.001) and between months (*F*_*11,893.51*_ = 7.16, *P* < 0.001), with a significant interaction between month and population (*F*_*32,891*_ = 3.09, *P* < 0.001). Individuals from the population S_italicus5 generally consumed more prey items than those from other populations (Table 2), while the largest number of consumed prey was generally observed in May, June, October and December (Fig. 4A). Individuals from population S_italicus4 significantly consumed less prey in May, October and December, while those from S_italicus6 significantly consumed less prey in May and September.

The diversity of prey items consumed (Shannon index) was significantly different between populations (*F*_*5,570.53*_ = 6.21, *P* < 0.001) and months of the year (*F*_*10,600.63*_ = 10.05, *P* < 0.001), and was also significantly affected by the interaction between these two factors (*F*_*31,595.09*_ = 2.52, *P* < 0.001). The diversity of prey consumed was generally lower in May, June and July (Fig. 4A), while individuals form S_italicus6 population showed the highest (Table 2). In November, individuals of S_italicus3 and S_italicus7 showed significantly higher prey diversity, while S_italicus4 and S_italicus6 showed the lowest in September and May, respectively.

**Figure 3:**
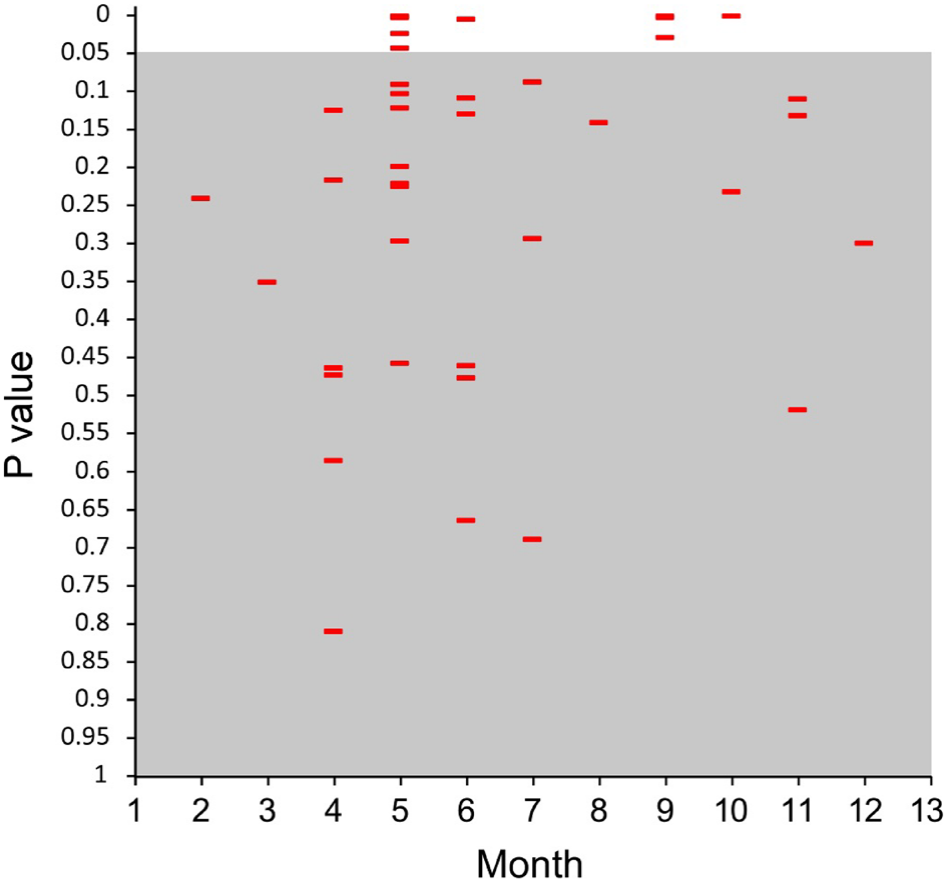
PERMANOVA analysis testing the effects of the factors “population” (fixed), “month” (fixed, repeated) and “individual” (random, nested within “population”) on *Speleomantes italicus* dietary habits: results of the pair-wise tests performed on the 46 possible combinations population/month. Noticeably, only16 were statistically significant (*P* < 0.05), and occurred in the months May–June and September–October.

The non-metric multidimensional scaling (NMDS) performed on the stomach content of salamanders highlighted a remarkable difference in the temporal pattern of trophic niche variation among the studied populations (Fig. 2). The PERMANOVA analysis supported this result: we detected a significant effect of both population and month of survey, and a strongly significant interaction between these two factors (Table 3). Pairwise comparisons further indicated that niche differences between populations were particularly strong in spring (May, June) and autumn (September, October; Fig. 3). Individual identity explained a limited amount of variation, indicating that our results are not affected by potential biases due to individual resampling (Table 3).

**Table 3:** Summary of the results of a PERMANOVA analysis testing the effects of the factors “population” (fixed), “month” (fixed, repeated) and “individual” (random, nested within “population” on *Speleomantes italicus* dietary habits.

Factor	df	SS	MS	Pseudo-F	P (perm)	P(MC)
Population (1)	2	8207.1	4103.5	3.1522	0.009	**0.002**
Month (2)	7	29,863	4266.1	3.3218	0.012	**0.001**
Individual (3)	491	6.29E+05	1280.4	0.96859	0.608	0.625
1 × 2**	16	40,392	2524.5	1.966	0.012	**0.001**
2 × 3**	68	87,235	1282.9	0.97049	0.571	0.612
Res	43	56,840	1321.9			
Total	636	1.17E+06				

In bold significant (*P* < 0.05) factors.

**Term with one or more empty cells.

**Figure 4:**
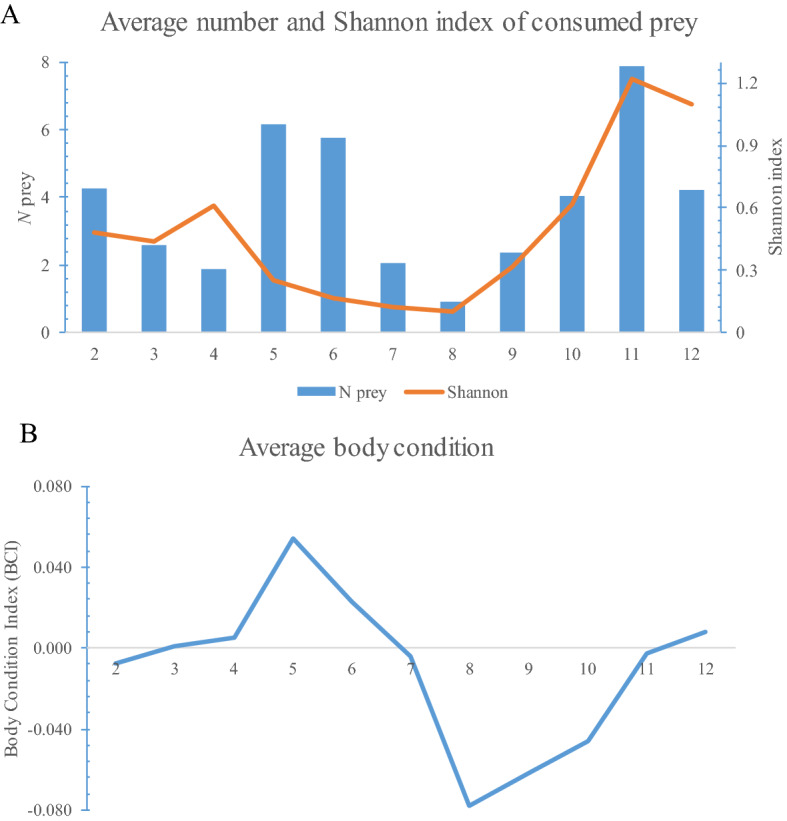
(**A**) Average number and the diversity (Shannon index) of consumed prey in the six populations of *Speleomantes italicus*. (**B**) Annual variation of body condition index (BCI) for the same populations. Numbers on x axis indicate the months; January is not included as no salamanders were captured.

The body condition index (BCI) of salamanders was significantly affected by sex (*F*_*2,67.88*_ = 3.93, *P* = 0.02), population identity (*F*_*5,547.78*_ = 10.63, *P* < 0.001), by the number of consumed prey (*F*_*1,485.51*_ = 23.86, *P* < 0.001), month (*F*_*11,586.55*_ = 3.59, *P* < 0.001), and by the interaction between population and month (*F*_*32,526.33*_ = 3.23, *P* < 0.001). BCI was lower in juveniles, while was higher in individuals that consumed more prey. The body condition of salamanders was generally higher in May, June, July and December (Fig. 4B). Individuals of the S_italicus4 population generally showed the lowest BCI, which was significantly lower during eight months (May, June, July, August, September, October, November, December) compared to the remaining months, while S_italicus7 individuals showed the highest BCI in November.

## Discussion

Our study provided the first evidence of year-round foraging activity in *Speleomantes italicus*. *Speleomantes* salamanders are particularly active during spring and autumn, when thermally mild and rainy conditions allow them to leave their shelters to forage also in surface environments^36,55^; for this reason, studies on their diet have mainly focused on these two seasons^41,42,56^. The microclimate of the deeper areas of caves, mines and other types of subterranean environments is less influenced by the external climate, as throughout the year there are constantly high humidity levels and relatively cold temperatures^47,57^. Ultimately, they represent a safe refuge for *Speleomantes* along with a multitude of other cave-dwelling species^50,58,59^. Most invertebrates found in subterranean environments are included in the *Speleomantes* diet^41,42,60^, which means that these salamanders do not necessarily have to leave these environments to forage; *Speleomantes* are in fact able to locate their prey in complete darkness^30^. In our study, *Speleomantes* had prey in their stomach (albeit with varying frequency; Table 2) in all the months where we found them, giving evidence that they forage all year round. January, the only month in which we did not find active salamanders, is probably the hardest month in terms of prohibitive climatic conditions for both *Speleomantes* and their potential prey. The most consumed prey are Diptera (flies), flying insects that often occur with high abundance in the areas surrounding the cave entrance^50,59^; in fact, this group represented about 70% of the overall diet of *S. italicus* (Table 2), and between 58 and 94% for the other species of *Speleomantes*^41,42,61,62^. During the warmer months the overall diversity of prey consumed by *S. italicus* decreased, favouring a higher proportion of Diptera (Table 2). Contextually, in this period also the number of consumed prey increased. When the external microclimate is too harsh (hot and dry), *Speleomantes* cannot leave their subterranean refuge^47^, while other surface species are moving underground to escape the unsuitable climate. This is, for example, the case of crane flies (Diptera), species that can reach very high densities when they move in subterranean environments^59^. These insects therefore become an easy target for *Speleomantes*, which can opportunistically prey upon a large number of prey without leaving the cave. This result is in agreement with previous analyses performed in summer and autumn, where a lower diversity of prey consumed (biased towards Diptera) was observed during the warmer period^16^.

**Figure 5:**
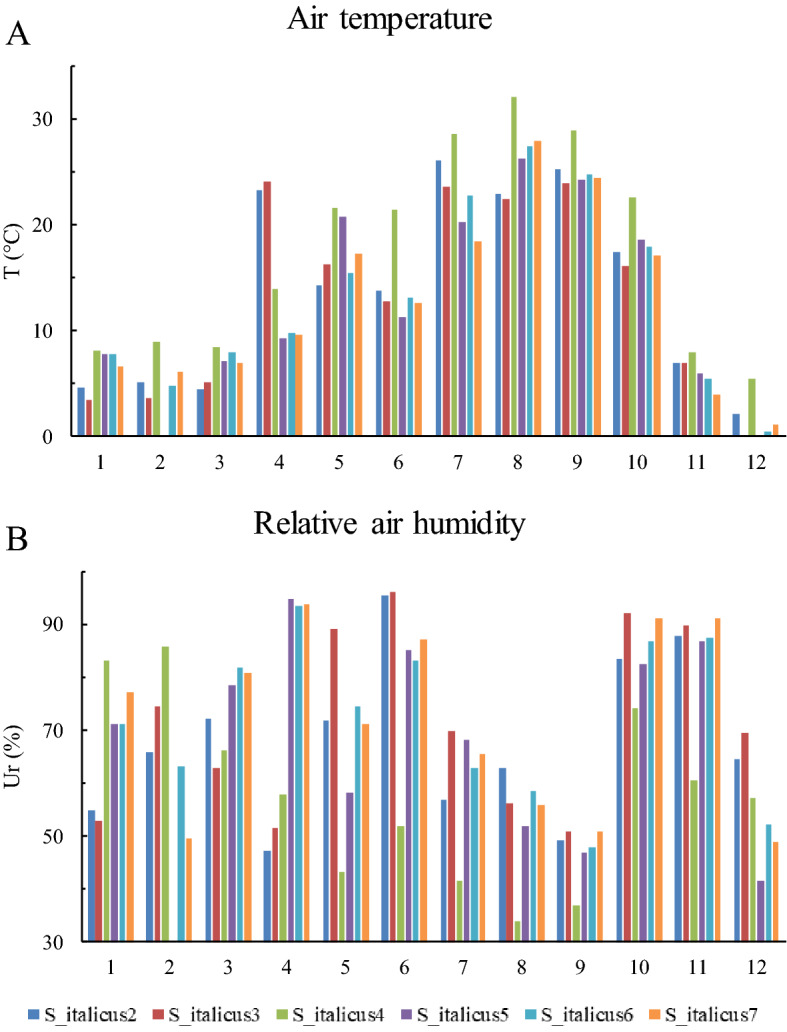
Microclimatic data recorded throughout a year nearby the entrance of the studied caves. Data on air temperature (**A**) and relative humidity (**B**) were monthly collected in a shaded area in the proximity (about 5 m) of the main entrance for each cave. Numbers on x axis indicate months. Data is retrieved from^47^.

Individuals from the population S_italicus5 were those consuming the larger *per capita* number of prey. This population showed the lowest estimated abundance of individuals (0.1 salamander/m^2^), a characteristic that may reduce the intraspecific competition, with a positive effect on individuals’ foraging success^54,63^. In this scenario, we would expect a better body condition of individuals from this population, and a corresponding lower body condition in the population S_italicus2 which has an abundance 50 times higher (Table 1); however, our analyses did not confirm this hypothesis. Interestingly, individuals from the population S_italicus5 were found in two months only, May and October (Table 2). Except for S_itailcus4, all the other five studied populations are characterized by comparable microclimatic conditions (Fig. 5), and therefore we would not expect a substantial variation in the foraging activity among them^36,47^. Additional factors not considered here may play an important role in affecting *Speleomantes* activity pattern. Consequently, the reasons behind the particular foraging activity observed in the population S_italicus5 remain unknown and definitely deserve further investigations.

The biological activities of subterranean *Speleomantes* populations, including foraging and reproduction, are strictly dependent on the external environmental conditions that occur in the surrounding area^36,64^. A previous analysis of outdoor microclimatic variation in these populations showed a high variability in terms of air temperature and relative humidity^47^ (see also Fig. 5), a condition that probably explains the divergence in foraging activity (i.e., number and diversity of prey consumed) observed in this study. For example, the population S_italicus4 is the only one located at an altitude < 500 m a.s.l. (Table 1), where microclimatic conditions were the warmest and the driest (Fig. 5), and likely sub-optimal^36^. Unsuitable external microclimatic conditions probably reduce the foraging in outdoor environments of *Speleomantes*^37,65^, where the prey abundance and diversity are the highest^36,50^. Indeed, individuals from S_italicus4 not only consumed less diverse and fewer prey (Table 2), but also showed the poorest body condition. Salamander body condition is strongly correlated with the quantity (and perhaps the quality) of the prey consumed^66,67^, therefore the lower foraging activity of the individuals of this population had an evident negative effect on their body condition^36^. Indeed, individuals from the population S_italicus7 showed the most diverse diet in November (Table 2), as well as the best body condition index. The relationship between microclimatic conditions, diet and body conditions confirms that sub-optimal microclimate affects multiple parameters of salamander populations^36^, and highlights the complex linkages between different dimensions of a species’ niche, such as trophic and climatic niche, explained here by the limited outdoor foraging in dry and warm environments.

The overall body condition of salamanders was highest before the periods when they strongly reduce their activity due to the highly unsuitable climatic conditions occurring in mid-summer and winter (Fig. 4B)^30^. Indeed, salamanders tend to consume as much prey as possible during the favourable seasons (i.e., spring and autumn), to withstand long periods of fasting in the deepest areas of caves, where prey is scarce but the microclimate is optimal^36,47,50^. However, the harsh climatic conditions occurring in summer and winter affect *Speleomantes* as well as their potential prey^58^; therefore, the temporal scarcity of prey may represent an additional factor promoting the reduced activity of these salamanders. Juveniles generally showed the lowest body condition. Young salamanders have physical constraints (i.e., limited mouth size) that force them to prey on smaller prey^16^ which might contribute little to increasing the salamander’s body mass. Furthermore, juveniles tend to accelerate their growth to reach sizes which allows them to become unsuitable target for some of their predators^68,69^. Consequently, most of the energy intake is devoted to increasing their size rather than their body mass^36,70^.

## Conclusions

With this study we have been able to answer some of the open questions related to the trophic niche of *Speleomantes*, for example whether they forage all year round, and how the foraging activity differ between conspecific populations. However, our results also paved the way for new further studies. First, *Speleomantes* are able to forage all year round and there is potential variability in feeding strategies among conspecific populations^54,71^. A more complete study on the annual diet of the different species is needed to understand the specific requirements of populations, and how diet variation affects populations dynamics^36^. Secondly, it may be interesting to evaluate whether the variation in *Speleomantes*’ seasonal diet is due only to the abundance and availability of prey, or whether an individual preference component is also included, for example towards the most profitable prey^72,73^. Third, a comparison with more strictly epigean populations (i.e., those that not use caves as shelters) would highlight the pros and cons of choosing to live in subterranean environments^57^. Finally, the body condition of *Speleomantes* is not constant, but changes according to the foraging activity adopted by the individuals of the different populations. This is important information that must to be taken into account when performing ecological studies or when aiming to assess the conservation status of species.

## Methods

### Dataset

We analysed the dataset published by Lunghi et al.^74^. This dataset collects dietary information on 495 individuals of *Speleomantes italicus* from six subterranean populations (i.e., inhabiting natural caves) distributed in three municipalities (2 populations in Prato, 1 in Vaiano, and 3 in Pistoia) (Table 1, Fig. 1). Despite the proximity of some populations (< 100 m straight line between two caves), the high site fidelity of this species allows them to be considered distinct populations, as confirmed by capture-mark-recapture studies^74,75^. These populations were surveyed inside the caves twice a month for a full year (May 2020–April 2021) during day-time (9 am–6 pm). Surveying multiple populations allows detecting potential inter-population variability in their trophic niche^54,71^. We estimated the maximum population density for each population as the ratio between the overall captured individuals and the surveyed area (Table 1). During each survey the captured *Speleomantes* were placed in disinfected plastic boxes until the end of the capture sessions (i.e., when the operator reached the end of the explored area of caves). *Speleomantes* were then weighted using a digital scale, photographed along a reference card^76^ and finally underwent the stomach flushing procedure, a non-lethal method that is widely adopted in studies on these protected species^28,41^. The identity of captured salamanders was assessed through the use of Visual Implant elastomers and natural dorsal pattern^75,77^. A total of 956 stomach flushing were performed (Table 2), with some salamanders sampled multiple times^74^. An interval of at least 10 days between two surveys ensured that recaptured individuals were not stomach-flushed too often. Prey items were recognised and counted following Lunghi et al.^41^; the dataset includes 31 different prey categories^74^.

### Data analysis

We used Generalized Linear Mixed Models (GLMMs) to assess the potential effects of environmental and biological factors on multiple features related to the trophic niche of *Speleomantes*. We first evaluated the potential drivers of the frequencies of empty stomach using a binomial GLMM where the dependent variable was the stomach condition (empty/full), while the independent variables were the snout-vent length (SVL, log-transformed) of salamanders, sex (female, male, juvenile), month of survey and population identity. We added as a further independent variable the interaction between month and population, as each population occurs at different elevation (Table 1), a condition that likely affects population phenology^36,47^. Salamander identity was used as random factor. We run two further GLMMs to evaluate the potential effects of the previously considered independent variables on both the number and diversity of consumed prey. As dependent variable, in the first model we used the log-transformed number of prey consumed, while in the second we used the diversity of prey items (Shannon index). Independent and random variables remained the same.

The vegan package^78^ was used in the R statistical environment (v. 4.1.2)^79^ to compute a (log + 1) transformation of salamanders’ dietary data and create a dissimilarity matrix using the Bray–Curtis dissimilarity index. To this end, the original dataset was reduced by eliminating individuals (*i*) with empty stomachs or having only unidentifiable prey in their stomachs; (*ii*) belonging to populations where < 3 individuals were observed within a month. The selection reduced the dataset to 637 stomach content records. The dataset included repeated observations, as some individuals were resampled both in the same month and in different months^74^. A non-metric multidimensional scaling (nMDS) plot was created from the Bray–Curtis dissimilarity matrix and used to visualize the temporal patterns of dietary variation across the 6 populations. At this stage, our aim was to illustrate overall time-dependent variations among the populations, thus nMDS results were provided as month-population centroids and no attempts were made to take into account repeated observations.

Variations in diet over time were compared among populations using a repeated measure permutational multivariate analyses of variance, PERMANOVA^80^ (vegan function *adonis,* 9999 permutations of the Bray–Curtis distance matrix), with Population as a fixed factor (6 levels), sampling month as a fixed repeated factor (12 levels). Individuals were a random factor nested within population in order to take into account inter-individual differences. PERMANOVA pair-wise tests were further used to determine in each month inter-population differences in dietary habits.

Finally, we assessed whether individual body condition was also affected by the same variables, as populations from different environments may exhibit different activity periods and likely diverge in their peak of body condition^36^. The body condition index (BCI) chosen was the residual index, widely adopted to evaluate the body condition in salamanders^81,82^. Residuals (i.e., the difference between observed and expected body mass) were estimated according to a log–log relationship between the total length of salamanders and their weight. A GLMM was subsequently run using the salamanders BCI as dependent variable, while together with the independent variables used in previous GLMMs we added the number of consumed prey as an additional variable. Random variables remained the same as in the previous GLMMs.

## Data Availability

Data are already published and publicly available, with those items properly cited in this submission. The full dataset can be found here https://esajournals.onlinelibrary.wiley.com/doi/epdf/10.1002/ecy.3641.
